# Triggering Receptor Expression on Myeloid Cells-1 (TREM1) Promoter Hypomethylation and Its Overexpression Associated With Poor Survival of Cancer Patients: A Pan-Cancer Analysis

**DOI:** 10.7759/cureus.64640

**Published:** 2024-07-16

**Authors:** Pinky Ruskin, Chandra Pandi, Balachander Kannan, Anitha Pandi, Smiline Girija A. S., Vijayashree Priyadharsini Jayaseelan, Paramasivam Arumugam

**Affiliations:** 1 Microbiology, Saveetha Dental College and Hospitals, Saveetha Institute of Medical and Technical Sciences, Saveetha University, Chennai, IND; 2 Molecular Biology Lab, Centre for Cellular and Molecular Research, Saveetha Dental College and Hospitals, Saveetha Institute of Medical and Technical Sciences, Saveetha University, Chennai, IND; 3 Clinical Genetics Lab, Centre for Cellular and Molecular Research, Saveetha Dental College and Hospitals, Saveetha Institute of Medical and Technical Sciences, Saveetha University, Chennai, IND

**Keywords:** therapeutic target, novel biomarker, dna methylation, hnscc, trem1

## Abstract

Background

Triggering receptor expression on myeloid cells-1 (TREM1) belongs to the immunoglobulin superfamily and is implicated in various conditions, including infectious and non-infectious diseases, autoimmune disorders, and cancer. Notably, TREM1 is significantly dysregulated in numerous cancer types. However, the underlying mechanism driving TREM1 mRNA expression in cancers remains unclear.

Objective

This study aims to analyze the promoter methylation level of TREM1 and its overexpression with cancer.

Methods

This study utilized The Cancer Genome Atlas (TCGA) cohort to analyze the methylation and expression levels of TREM1 in cancers. The University of ALabama at Birmingham CANcer (UALCAN) database facilitated data analysis from the TCGA dataset. Additionally, survival analysis was conducted using the TCGA dataset via Kaplan-Meier (KM) plots to identify significant associations with prognosis.

Results

Promoter methylation analysis revealed that TREM1 is hypomethylated in cancers, resulting in significantly overexpressed mRNA across various cancer types. This methylation and expression showed a negative correlation. Furthermore, high TREM1 mRNA expression was linked to poor prognosis in several cancers.

Conclusion

TREM1 gene expression negatively correlates with promotor DNA methylation and is associated with poor survival. It may serve as a prognostic marker and biomarker for various cancers. Future research should focus on further validation and antitumor immunity to elucidate its oncogenic role in cancers.

## Introduction

Cancer remains one of the most devastating diseases of the 20^th^ and 21^st^ centuries. In 2022, 20 million new cases and 9.7 million deaths were documented worldwide, and exposure to various carcinogens has become increasing. Cancers can develop from any organ or body structure and are made up of microscopic cells that have lost the ability to stop growing [1]. Cancerous cells transition from normal cells due to the failure of the body's immune cells to detect and eliminate newly formed cancer cells when they are present in small numbers [2]. Genetic and epigenetic alternations also play a major role in cancer development. In genetic alternations, missense mutations, insertions, deletions, copy number variations, and recombination of DNA are involved [3]. Oncogenes and other inflammatory genes play a major role in tumor development and are involved in tumor microenvironment. Our previous study identified genes playing a major role in cancer development and prognosis [4-6]. Furthermore, gene-environment interactions disrupt the tumor microenvironment, leading to uncontrolled cell proliferation. Cancer cells acquire traits for extended survival and abnormal growth, bypassing normal regulatory mechanisms. Understanding these interactions is crucial for developing targeted therapies that selectively treat cancer cells while sparing normal cells [7].

Triggering receptors expressed on myeloid cells (TREM) is a family comprising five subtypes - TREM1 to TREM5 - and belongs to the immunoglobulin superfamily. These receptors are prominently expressed in bone marrow-derived cells and are known as activated receptors [8]. The different types of TREM protein within the family play distinct roles in inflammatory responses [9]. One of this family's most well-studied members, TREM1, is essential for regulating immune responses in myeloid cells. The activating receptor protein TREM1, which is expressed by monocytes and neutrophils, initiates a series of intrusive cellular events, such as the release of cytokines, neutrophil degranulation, and phagocytosis upon engagement with the cell membrane [10]. TREM1 is a potent amplifier of pro-inflammatory immune responses. Accumulating evidence suggests that TREM1 plays a crucial role in chronic and non-infectious inflammatory disorders, including various types of cancer [11]. In inflammatory disorders such as pneumonia, inflammatory bowel disease, intraperitoneal infection, and others, there is an increase in the levels of TREM1 [12,13]. When TREM1 is activated, it triggers the expression of various cytokines such as tumor necrosis factor-alpha (TNF-α), macrophage colony-stimulating factor (M-CSF), interleukin-1 alpha (IL-1α), interleukin-1 beta (IL-1β), interleukin-6 (IL-6), monocyte chemoattractant protein-1 (MCP-1), and TREM1 itself. This activation amplifies the inflammatory response, particularly during inflammation. Studies conducted in vitro have demonstrated that inhibiting TREM1 can effectively impede the invasion of cancer cells [14]. Furthermore, TREM1 was shown to be overexpressed in some solid tumors, and higher TREM1 levels appear to be related to a poor tumor prognosis [15,16]. In our previous study, we elucidated the significance of TREM1 expression in both head and neck squamous cell carcinoma (HNSCC) and oral squamous cell carcinoma (OSCC) [6]. However, the high expression of TREM1 in cancer mechanisms is still unclear.

We hypothesized that DNA alternation may influence mRNA and protein expression in cancer. Particularly, epigenetic alternation significantly alters gene expression. Epigenetics is the inheritance of information based on gene expression rather than base sequence. Epigenetic processes such as DNA methylation, histone tail modifications, nucleosome placement, and noncoding RNA are critical for preserving heritable changes in gene expression potential and chromatin architecture over cell generations. DNA methylation is now recognized as a major mechanism in the regulation of gene expression in this manner, and evidence for its significance in the development of a wide range of malignancies is accumulating [17,18]. The relation between DNA methylation and TREM1 remains unclear. Our research aims to investigate the correlation between TREM1 gene expression and DNA methylation. We are specifically analyzing the association of methylation promoters related to cancer while also focusing on uncovering the relationship between high expression of the TREM1 gene and methylation.

## Materials and methods

Gene expression analysis by The Cancer Genome Atlas (TCGA) dataset analysis

In this study, we examined the expression of the TREM1 gene across various cancers using The Cancer Genome Atlas database (TCGA). Using the TCGA dataset, researchers can access Level 3 RNA-seq data, enabling analysis of gene expression and survival outcomes for approximately 20,500 protein-coding genes across 33 distinct tumor types. TCGA includes primary untreated tumors with matched normal samples, processed by the Biospecimen Core Resource (BCR) to ensure consistent pathology assessment and high-quality molecular analytes (DNA and RNA). Samples are reviewed by a pathologist, requiring at least 80% tumor nuclei and less than 20-30% necrotic tissue, followed by nucleic acid isolation and genotyping. DNA methylation data have been harmonized using the SEnsible Step-wise Analysis of DNA MEthylation BeadChips (SeSAMe) pipeline and are available at the Genomic Data Commons (GDC) Data Portal. TREM1 expression and promoter methylation levels were assessed using these high-quality analytes, which underwent rigorous molecular quality control before genomic analysis. All procedures comply with human subjects protection guidelines, and informed consent, by TCGA. The University of ALabama at Birmingham CANcer (UALCAN), a tool commonly utilized for such analyses, provides a platform to perform these investigations. Specifically, we analyzed the mRNA expression of TREM1 in pan-cancer samples, correlating it with methylation studies through the TCGA-UALCAN database (http://ualcan.path.uab.edu/analysis.html) [19]. 

Survival analysis by Kaplan-Meier (KM) plotter

We conducted survival analysis across various cancers, correlating it with the TREM1 gene expression. For this analysis, we utilized the Kaplan-Meier (KM) plotter database (http://kmplot.com/analysis) [20], which uses the data from the TCGA dataset. The TCGA project provides comprehensive genomic and clinical data, including gene expression and overall survival (OS) information. To analyze TREM1 gene expression, the KM plotter used the RNA-seq data and OS data for relevant patients. Patients were stratified into high and low TREM1 expression groups based on a defined cut-off value. This cut-off was derived using statistical software like R (R Foundation for Statistical Computing, Vienna, Austria), employing packages such as 'survival' and 'survminer' to optimize the separation of survival curves in KM plots. The KM plot visually represents survival probabilities over time, and the log-rank test was used to assess the significance of differences between high and low-expression groups.

Statistical analysis

IBM SPSS Statistics for Windows, Version 25 (IBM Corp., Armonk, United States) was used for statistical analysis using the Student’s t-test or one-way analysis of variance (ANOVA) and log-rank test. Results are presented as the mean and standard deviation of three independent experiments. Statistical significance was set at p<0.05. 

## Results

Hypomethylation and high expression of TREM1 are associated with various cancers

**Figure 1:**
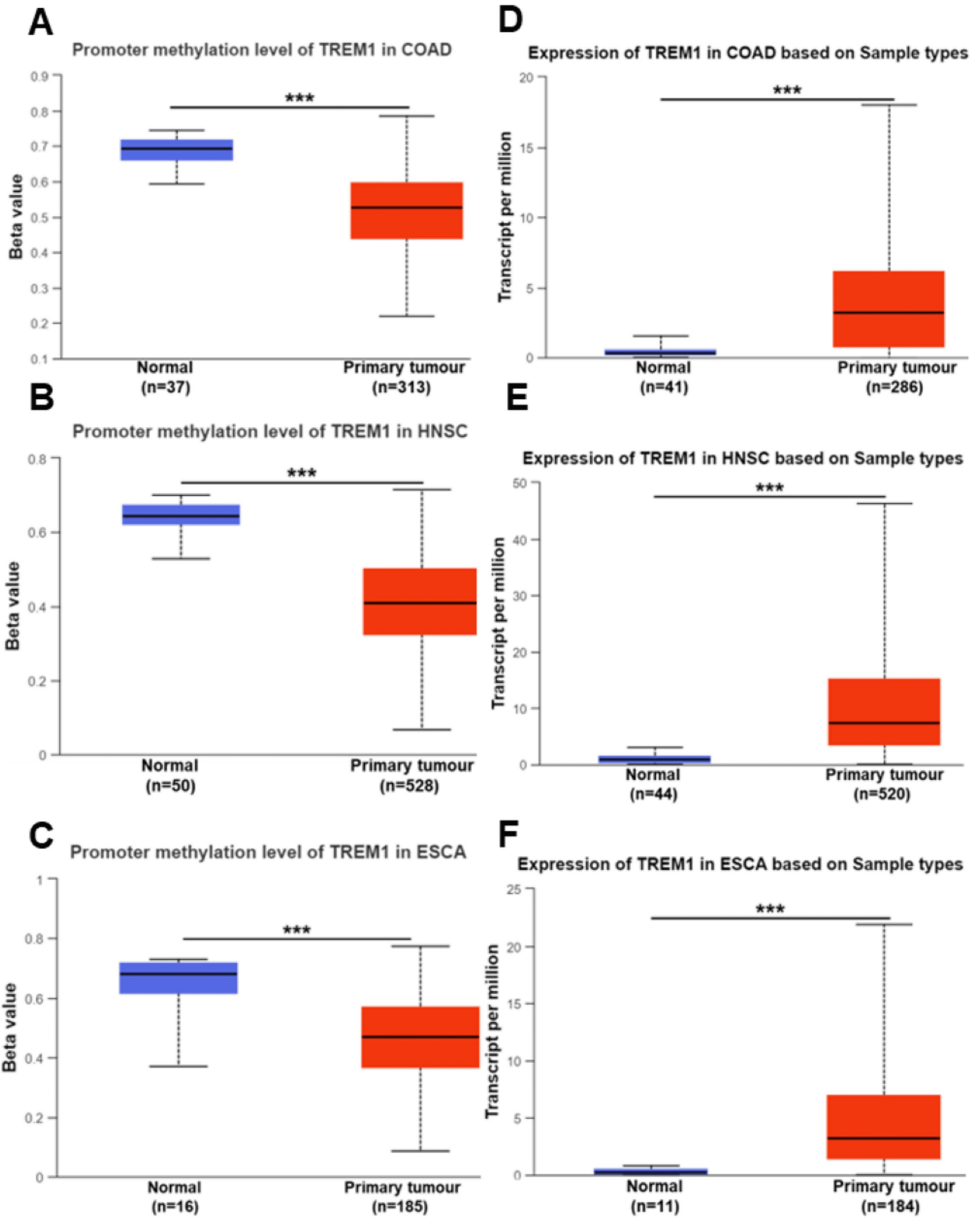
TREM1 methylation and expression in various cancers. (A-C) TREM1 promoter methylation level in COAD, HNSC, and ESCA based on the TCGA cancer and normal samples. (D-F) TREM1 mRNA expression level in COAD, HNSCC, and ESCA based on the TCGA cancer and normal samples. The blue color bar indicates the normal samples and the red color indicates the tumor samples. TREM1: Triggering receptor expression on myeloid cells-1; COAD: Colon adenocarcinoma; HNSC: Head and neck cancer; ESCA: Esophageal cancer; TCGA: The Cancer Genome Atlas

**Figure 2:**
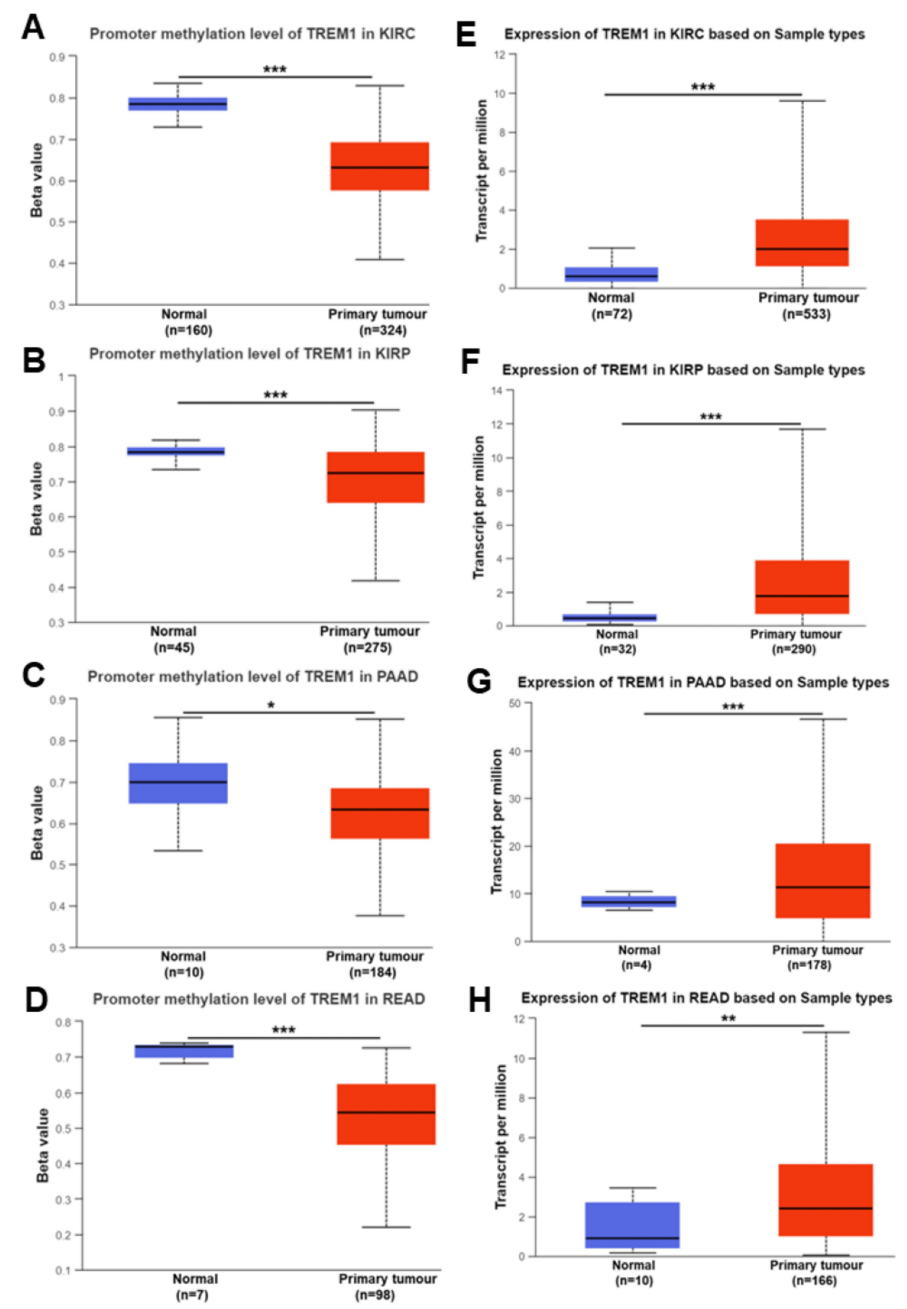
TREM1 methylation and expression in various cancers. (A-D) The promoter methylation level of TREM1 in KIRC, KIRP, PAAD, and READ in tumor and normal samples. (E-H) TREM1 expression level in KIRC, KIRP, PAAD, and READ based on sample types in tumor and normal samples. The blue color bar indicates the normal samples and the red color indicates the tumor samples. TREM1: Triggering receptor expression on myeloid cells-1; KIRC: Kidney renal cell carcinoma; KIRP: Kidney renal papillary cancer; PAAD: Pancreatic adenocarcinoma; READ: Rectum adenocarcinoma

**Figure 3:**
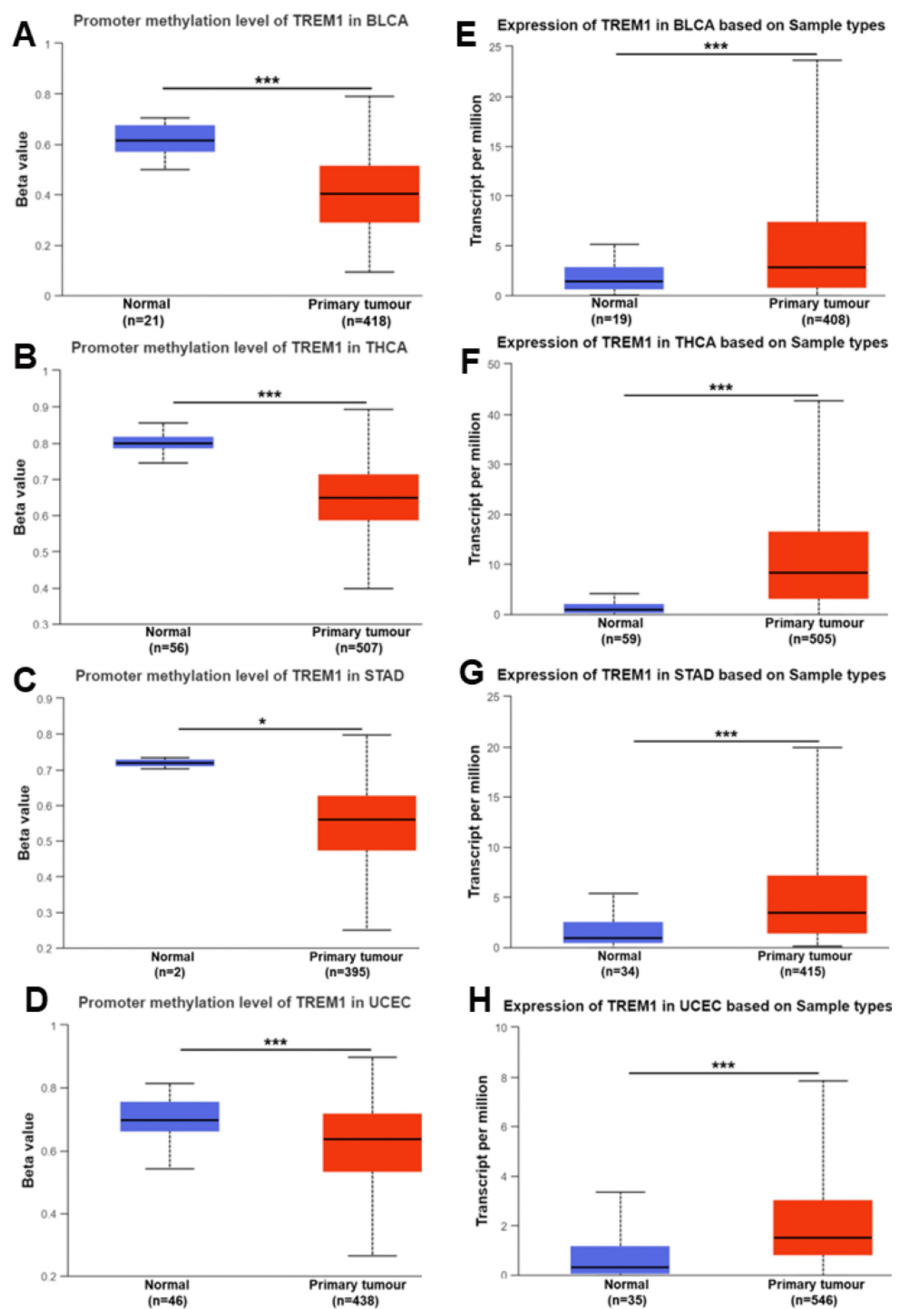
TREM1 methylation and expression in various cancers. (A-D) The promoter methylation level of TREM1 in BLCA, THCA, STAD, and UCEC in tumor and normal samples. (E-H) TREM1 mRNA expression level in BLCA, THCA, STAD, and UCEC based on the tumor and normal samples. The blue color bar indicates the normal samples and the red color indicates the tumor samples. TREM1: Triggering receptor expression on myeloid cells-1; BLCA: Bladder cancer; THCA: Thyroid carcinoma; STAD: Stomach adenocarcinoma; UCEC: Uterine corpus endometrioid carcinoma

In our study, we examined the correlation between promoter methylation and TREM1 gene expression across various cancers using the TCGA dataset. We observed a significant decrease in the promoter methylation level of TREM1, suggesting hypomethylation, in several cancers compared to normal samples. Specifically, this trend was evident in colon adenocarcinoma (COAD), head and neck cancer (HNSC), and esophageal cancer (ESCA) (Figures 1A-1C, P<0.001), as well as in kidney renal papillary cancer (KIRP), kidney renal cell carcinoma (KIRC), pancreatic adenocarcinoma (PAAD), and rectum adenocarcinoma (READ) (Figures 2A-2D, P<0.05), and in bladder cancer (BLCA), thyroid carcinoma (THCA), stomach adenocarcinoma (STAD), and uterine corpus endometrioid carcinoma (UCEC) (Figures 3A-3D, P<0.05). Conversely, TREM1 gene expression was significantly upregulated in tumor samples compared to normal samples across various cancers. This upregulation was observed in COAD, HNSC, and ESCA (Figures 1D-1F, P<0.001), as well as in KIRC, KIRP, PAAD, and READ (Figures 2E-2H, P<0.01) and in BLCA, THCA, STAD, and UCEC (Figures 3E-3H, P<0.001).

Correlation between TREM1 expression and prognosis

**Figure 4:**
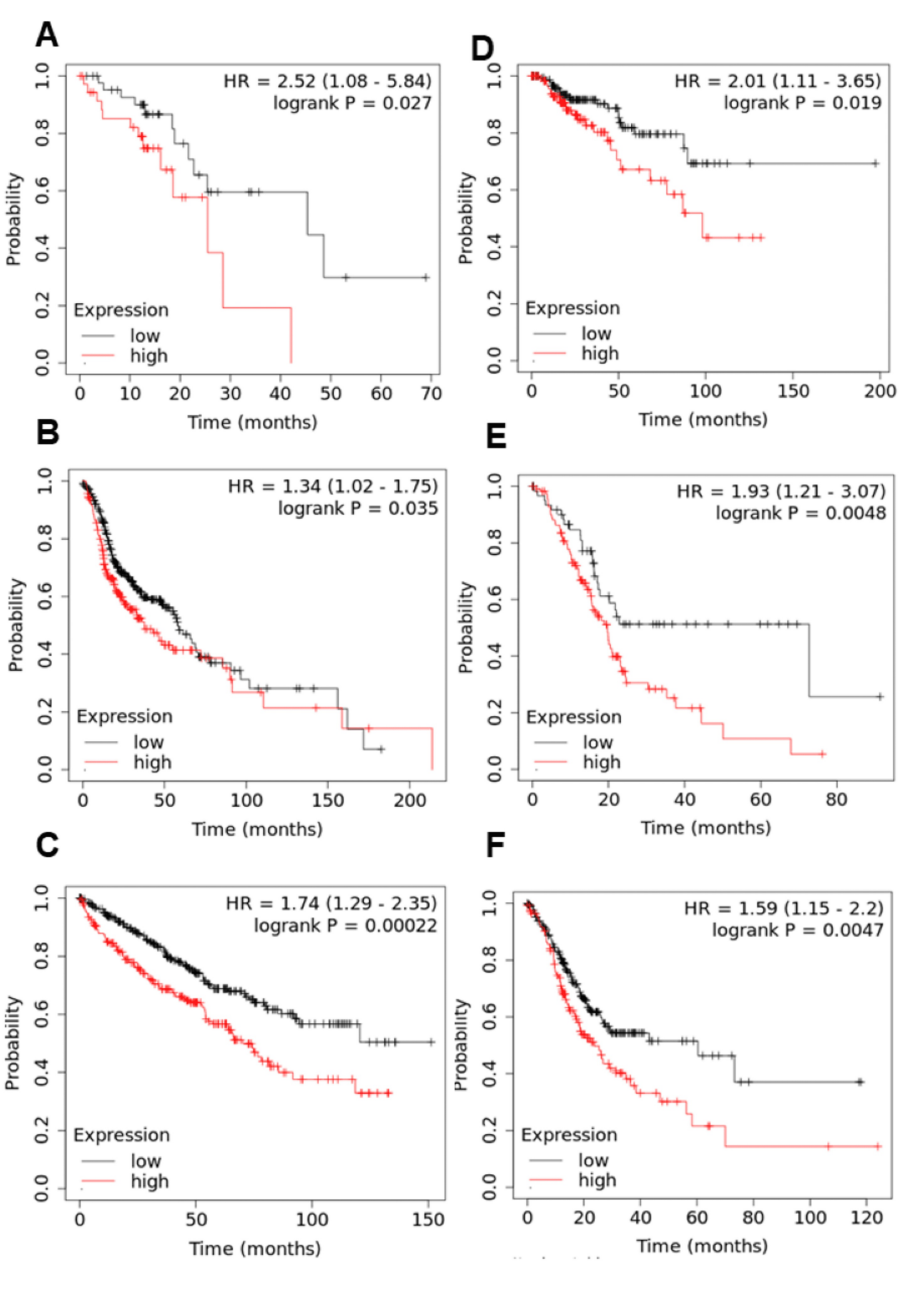
Survival analysis of the TREM1 gene in several cancers such as (A) ESCA, (B) HNSC, (C) KIRC, (D) KIRP, (E) PAAD, and (F) STAD. The TREM1 gene expression is classified into high and low, based on the cut-off value. The red color line indicates high TREM1 expression and the blue color line indicates low TREM1 expression. TREM1: Triggering receptor expression on myeloid cells-1; ESCA: Esophageal cancer; HNSC: Head and neck cancer; KIRC: Kidney renal cell carcinoma; KIRP: Kidney renal papillary cancer; PAAD: Pancreatic adenocarcinoma; STAD: Stomach adenocarcinoma

We further analyzed the prognosis rate of ESCA, HNSC, KIRC, KIRP, PAAD, and STAD cancers associated with TREM1 gene expression (Figures 4A-4F, P<0.05). TREM1 gene expression was classified into low and high based on the cut-off value, which was retrieved from the KM plot. It confirms that high TREM1 gene expression is negatively associated with overall survival rates, suggesting poor prognosis for these cancers (Table 1). 

**Table 1:** TCGA data: Hypomethylation in the TREM1 gene is associated with high expression and poor prognosis in different types of cancer. NS: Not significant; TCGA: The Cancer Genome Atlas; TREM1: Triggering receptor expression on myeloid cells-1

Cancer name	TCGA ID	Methylation	Expression	Prognosis
Bladder cancer	BLCA	Hypomethylation	High	NS
Colon adenocarcinoma	COAD	Hypomethylation	High	NS
Esophageal carcinoma	ESCA	Hypomethylation	High	Poor
Head and neck squamous cell carcinoma	HNSC	Hypomethylation	High	Poor
Kidney renal clear cell carcinoma	KIRC	Hypomethylation	High	Poor
Kidney renal papillary cell carcinoma	KIRP	Hypomethylation	High	Poor
Pancreatic adenocarcinoma	PAAD	Hypomethylation	High	Poor
Rectum adenocarcinoma	READ	Hypomethylation	High	NS
Thyroid carcinoma	THCA	Hypomethylation	High	NS
Stomach adenocarcinoma	STAD	Hypomethylation	High	Poor
Uterine corpus endometrioid carcinoma	UCEC	Hypomethylation	High	Poor

## Discussion

The findings from our study illuminate the prevalent hypomethylation of TREM1 in multiple cancer types, coupled with increased gene expression levels, as evident from TCGA dataset analysis, suggesting a potential mechanism driving TREM1 dysregulation in tumorigenesis. Furthermore, elevated TREM1 expression correlates significantly with poor prognosis in ESCA, HNSC, KIRC, KIRP, PAAD, and STAD cancers, as indicated by KM plot analysis, highlighting its potential as a prognostic marker. These findings underscore the importance of further investigating TREM1-associated pathways and their therapeutic implications in cancer management, offering promising insights into personalized treatment strategies to enhance patient outcomes.

Cancer is a leading cause of death around the world. Early detection and treatment of cancer are crucial for improved health management. The cancer evaluation is conducted including the nature of cancer, risk assessment, prevention, and health management [21]. Epigenetic changes, such as DNA methylation and histone modification, are integral processes involved in cancer development, contributing significantly to tumorigenesis. However, TREM1 involvement in cancers and its correlation with DNA methylation levels remain uncertain. In this study, we identified a significant relationship between hypomethylation and increased gene expression of TREM1 in the development of cancer.

Recent research findings have shed light on the pivotal role that TREM1 expression plays in inflammation-mediated carcinogenesis [10,14]. Prominently, elevated levels of TREM1 in macrophage-infected human malignancies, in conjunction with reduced levels of spontaneous TREM1 conformation, have been linked to tumor aggressiveness, recurrence, and inadequate patient survival. The involvement of TREM1 in inducing an increased inflammatory response has been linked to various cancer types, such as prostate, breast, colon, cervical, hepatocellular, lung, and renal cancers [11,22-26]. Certain other inflammatory disorders such as pancreatitis, gastric ulcers, psoriasis, ulcerative colitis, and vasculitis situations when TREM1 may be over-expressed [9,27-29]. Our previous research study suggests that the TREM1 gene is significantly overexpressed in HNSCC and OSCC [6]. Likewise, the current research study confirms that, based on the sample types, TREM1 shows elevated expression in various tumor tissue samples compared with normal tissue samples. 

As an epigenetic mechanism, the complex regulation of DNA methylation is essential for the initiation, maintenance, and progression of cancer. Effective management of DNA methylation is not only essential for regulating gene transcription but also extends to preserving genome integrity and influencing immune response modulation [17]. The initial methylation alteration identified in cancer was CpG (cytosine guanine dinucleotide) hypomethylation. Subsequent research studies have revealed that 5mC (5-methyl cytosine) content decreased in various cancer types [30]. Hypomethylation frequently occurs in benign hyperplasia and can serve as an early precursor in tumorigenesis. As tumors progress, there is a notable increase in the loss of methylation, with metastatic lesions exhibiting even higher levels of demethylation compared to primary tumors [31]. Likewise, our research study revealed a negative correlation between TREM1 gene expression and DNA methylation across various cancers. In tumor samples, we observed a downregulation of promoter methylation levels compared to normal samples, suggesting that this hypomethylation condition may play a role in tumorigenesis. Several research studies have identified DNA methylation as a promising biomarker for cancer. O^6^-methylguanine-DNA methyltransferase (MGMT) stands out as one of the first DNA methylation cancer biomarkers. Similarly, we propose that the hypomethylation status of TREM1 could also serve as a potential biomarker for various cancers. 

Numerous studies have examined the relationship between TREM1 gene expression and cancer survival rates. According to Ho et al., patients with non-small cell lung carcinoma (NSCLC) who have high expression of TREM1 in their tumor-associated macrophages are more likely to experience cancer recurrence and poor survival may have additional clinical implications [32]. Additionally, in a mouse xenograft NSCLC model, TREM1 inhibition suppressed NSCLC tumor growth. Likewise, a separate study demonstrated that TREM1 plays a significant role in influencing the growth, progression, and prognosis of hepatocellular carcinoma (HCC) and lung cancer [25,26]. Similarly, our study findings reveal that TREM1 expression is inversely correlated with patient survival rates. Moreover, targeting TREM1 could be a more effective therapeutic strategy for the treatment of various cancers.

Several TREM1 inhibitors have been developed, including the inhibitory peptides LP17 and LR12 and a TREM1-specific antibody, which block the receptor's ligand binding. However, the exact nature of TREM1's ligand(s) remains unclear, complicating clinical application. Studies have shown that ligand-independent inhibitors like LSKSLVF and GLLSKSLVF effectively reduce pro-inflammatory cytokine production and improve survival in experimental models [33]. Accumulating results suggest that targeting TREM1 could be a promising therapeutic strategy for cancer treatment.

TREM2-TREM5 are additional members of the TREM family with potential roles in tumorigenesis. TREM2 has been linked to cancer and neurodegeneration, with therapies targeting TREM2 showing promise in these areas. Research on TREM3 is limited due to its absence in humans, making it more relevant in mouse models. TREM4 and TREM5, though less studied, might also influence cancer progression and inflammatory diseases, warranting further investigation to elucidate their ligands, signaling pathways, and therapeutic potential in various disease models, including cancer [34].

This study has several limitations. We primarily established the association between DNA methylation and TREM1 expression through in-silico analyses. To bolster our findings, it is imperative to undertake validation studies, particularly focusing on gene expression and protein expression analyses using tumor and normal tissue samples via reverse transcription-quantitative polymerase chain reaction (RT-qPCR) and western blot experiments. It's worth noting that our in-silico study was conducted with a limited number of samples, necessitating further investigations with larger sample sizes. Additionally, future avenues for exploration include in-vivo and in-vitro studies. These additional investigations will provide deeper insights into the role of DNA methylation and TREM1 expression in carcinogenesis. 

## Conclusions

In our study, we focused on the relationship between TREM1 gene expression and DNA methylation in cancer development. We found that TREM1 gene expression is higher in various cancers while DNA methylation is lower. These findings suggest that increased TREM1 expression is associated with reduced DNA methylation, potentially promoting cancer growth. Additionally, we observed that high TREM1 expression is linked to poorer prognosis. This highlights TREM1 as a potential biomarker, prognostic indicator, and treatment target in cancer care. Further research is needed to understand TREM1's role better and develop new therapies for improved patient outcomes.
